# The impact of therapeutic landscapes on the mental health of older adults: a systematic review

**DOI:** 10.3389/fpsyg.2026.1898757

**Published:** 2026-08-20

**Authors:** Yan Han, Bin Li, Yuxin Hou, Juan Zhang

**Affiliations:** 1 Department of Spatial Culture Design, Graduate School of Techno-Design, Kookmin University, Seoul, Republic of Korea; 2 Faculty of Human Ecology, Universiti Putra Malaysia, Serdang, Selangor, Malaysia; 3 Department of Industrial Design, College of Art and Design, Beijing University of Technology, Beijing, China

**Keywords:** therapeutic landscapes, mental health, older adults, health benefits, systematic review

## Abstract

Therapeutic landscapes, as an important form of health-promoting environmental intervention, provide a feasible approach to improving the mental health of older adults. However, their modes of action and underlying mechanisms require further clarification. Following the PRISMA guidelines, this study systematically searched Web of Science, Scopus, JSTOR, ScienceDirect, PubMed, and PsycINFO. Studies were selected according to the PICOS framework and assessed using the Crowe Critical Appraisal Tool (CCAT) and the JBI Critical Appraisal Tools for risk of bias. Ultimately, 21 studies were included in this systematic review of the evidence on the effects of therapeutic landscapes on older adults’ mental health. The findings showed that therapeutic landscapes had positive effects on older adults’ mental health, primarily by alleviating depression, anxiety, stress, and loneliness and enhancing psychological well-being. However, these health effects did not occur uniformly but were jointly influenced by factors such as spatial accessibility, facility suitability, activity organization, seasonal and climatic conditions, and individual abilities. Further analysis revealed that different types of therapeutic landscapes had differential effects on older adults’ mental health. Nature-based landscapes focused primarily on landscape exposure and restorative experiences, structured intervention landscapes emphasized active participation, and sociocultural experiential landscapes highlighted social interaction and emotional support. This study systematically synthesized the evidence on the role of therapeutic landscapes in promoting older adults’ mental health, providing a reference for the theoretical development and practical application of therapeutic landscape research. Future studies should strengthen the empirical evidence derived from diverse regions and sample types, pay greater attention to the differing needs of various groups of older adults, and further investigate the pathways through which different types of therapeutic landscapes influence older adults’ mental health.

**Systematic review registration:**

https://inplasy.com/inplasy-2026-5-0126/, INPLASY202650126.

## 1 Introduction

Population aging is a common challenge currently faced worldwide. According to the World Population Ageing 2023 report released by the United Nations, the global population aged 65 years and above is projected to account for 16% of the total global population by the middle of this century (United Nations, 2024). Health, as a long-standing common pursuit of humankind, has received increasing attention in the context of population aging. However, health does not merely refer to the absence of disease and frailty at the physiological level; rather, it represents a complete state that integrates psychological and social dimensions. Therefore, mental health is also an important dimension for assessing the quality of life of older adults (World Health Organization, 1948). However, mental health problems among older adults are often insufficiently recognized and treated. Moreover, although many older adults appear physically healthy, they remain at risk of mental health problems, including depression and anxiety disorders. Survey evidence indicates that approximately 14.1% of adults aged over 70 years suffer from mental disorders (Institute for Health Metrics and Evaluation, 2024). In addition, the 2021 Global Health Estimates indicate that approximately one-sixth of suicide deaths worldwide occurred among older adults aged 70 years and above (World Health Organization, 2024).

In recent years, with increasing societal attention to the mental health of older adults, a growing body of research has emerged. Common mental health problems among older adults mainly include depression, anxiety, stress, loneliness, and social isolation. Depression is one of the most common mental disorders among older adults. It may not only lead to a loss of willingness to live and induce physiological diseases, but also reduce the effectiveness of treatment for physiological diseases and, in severe cases, may increase the risk of suicide (Mawar et al., 2018). Anxiety disorders are also prevalent. They can impair older adults’ functional abilities and affect their overall quality of life (Yu et al., 2016). Stress presents a high degree of heterogeneity. Existing studies have shown that 5% to 50% of older adults experience stress in daily life. Meanwhile, due to the widespread presence of stress, perceived stress is expected to increase multiple times over the next decade (Issalillah and Aisyah, 2022; Ansari and Kang, 2019). In addition, although loneliness and social isolation are not strictly defined as mental disorders, the negative experiences arising from a lack of social connection can have profound effects on health (Prohaska et al., 2020). Although the mental health of older adults has gradually become a major issue of societal concern, many older adults have difficulty accessing and continuously using mental health services over the long term due to differences in economic capacity, medical resources, and health literacy. This problem is particularly pronounced in resource-limited rural areas and grassroots communities.

In response to these challenges, environmental interventions represented by therapeutic landscapes provide a practical and feasible alternative pathway for promoting the mental health of older adults. The core concept of therapeutic landscapes emphasizes the use of specific natural environments to support health improvement. It advocates the use of the multidimensional characteristics of landscape spaces to help individuals reduce stress and restore attention, thereby reducing or alleviating the occurrence of psychological symptoms and related disorders (Gesler, 1992; Ulrich, 1983; Kaplan and Kaplan, 1989). For example, Ulrich first experimentally demonstrated in 1984 that patients with a view of the outdoor landscape from their windows had shorter hospital stays and used fewer analgesics (Ulrich, 1984). Comparative research on natural and urban stimuli also indicated that natural stimuli can more rapidly facilitate emotional recovery (Ulrich et al., 1991). Other scholars have further pointed out that greater amounts of green space around residential areas are associated with lower perceived stress and healthier cortisol secretion patterns (Roe et al., 2013). Clinical evidence has also shown that interventions involving natural environments and nature-based activities can significantly improve anxiety, depression, and stress (Jessen et al., 2025).

Over the past decade, research on therapeutic landscapes has experienced sustained growth (Yang et al., 2025), and the effects of therapeutic landscapes on the mental health of older adults have increasingly become a focus of scholarly attention. However, existing studies are dispersed across diverse settings and tend to focus on individual therapeutic landscape interventions, while a systematic review and synthesis of the evidence in this field is lacking. Therefore, this study examines relevant studies published between 2015 and 2025, systematically synthesizes the evidence on the mental health benefits of therapeutic landscapes for older adults, and addresses the following research questions (RQs):

RQ1: What are the general characteristics of studies on therapeutic landscapes and the mental health of older adults from 2015 to 2025?

RQ2: What measurement instruments and therapeutic landscape intervention approaches have been used to examine the effects of therapeutic landscapes on the mental health of older adults?

RQ3: How do therapeutic landscapes affect the mental health of older adults?

Overall, this study aims to examine the effects of different types of therapeutic landscapes on the mental health of older adults. Specifically, it analyzes the geographical distribution, temporal characteristics, and research methods of the included studies, systematically summarizes the measurement instruments and intervention approaches used in these studies, and further discusses the effects and potential pathways of action associated with different types of therapeutic landscapes.

## 2 Methods and materials

This study followed the PRISMA 2020 guidelines for systematic reviews (Page et al., 2021). The review protocol has been registered on the International Platform of Registered Systematic Review and Meta-analysis Protocols, with the registration number INPLASY202650126.

### 2.1 Search strategy

This study systematically searched six literature databases: Web of Science, Scopus, JSTOR, ScienceDirect, PubMed, and PsycINFO. The search was completed on October 19, 2025. These databases collectively provide broad coverage of relevant research in environmental science, landscape studies, public health, gerontology, psychology, and mental health. During the search process, the Boolean operators AND and OR were used in combined with keywords, and harmonized search terms and search strings were consistently used on each database to ensure consistency in the content searched across all databases (Table 1; Supplementary Table S1).

**Table 1:** Search concepts and corresponding search strings.

Search builder	Search string
Therapeutic landscapes	“therapeutic landscapes” OR “healing garden”
Mental health	“mental health” OR “psychological health” OR “psychological well-being” OR “anxiety” OR “depression” OR “stress” OR “loneliness”
Older adults	“older adult” OR “older adults” OR “older person” OR “older persons” OR “older people” OR “elderly”

### 2.2 Inclusion and exclusion criteria

To systematically define the research question and the scope of the review and to improve the transparency and reproducibility of the study selection process, this study used the PICOS framework to establish the eligibility criteria (Table 2). The framework comprises five elements: Population, Intervention/Exposure, Comparison, Outcome, and Study Design, and provides relatively comprehensive coverage of the core components addressed in this review (Methley et al., 2014). Studies were eligible for inclusion only if they met the following criteria: (1) the study population consisted of older adults from any country or region who were aged 60 years or older, or participants identified by the original study as an older population using terms including, but not limited to, “older adults,” “older persons,” and “older people”; (2) the study involved therapeutic landscapes or related nature-based environmental interventions or exposures involving health-promoting landscape designs, including, but not limited to, therapeutic gardens, healing gardens, community green spaces, and green spaces associated with residential care facilities for older adults; (3) the study included comparisons between different exposure levels or different types of therapeutic landscapes, or adopted a within-group pre–post intervention design; (4) the study examined the mental health of older adults,including emotional or psychological outcomes such as depressive symptoms, anxiety, perceived stress, loneliness, and psychological well-being; and (5) the study was a peer-reviewed empirical study employing quantitative, qualitative, or mixed-methods research.

**Table 2:** PICOS criteria for inclusion of studies.

Items	Detailed inclusion criteria
Population	Older adults from any country or region (aged ≥60 years)
Intervention	Therapeutic landscapes, healing landscapes, restorative environments, and related settings
Comparison	Nature-based interventions, different levels of green space exposure, usual care, and pre–post comparisons (a control group was not required for qualitative studies)
Outcome	Psychological states, including depression, anxiety, stress, loneliness, and psychological well-being
Study design	Quantitative, qualitative, and mixed-methods studies

The exclusion criteria were as follows: (1) books, book reviews, conference papers, research reports, blogs, web pages, news articles, magazine articles, theses and dissertations, review articles, preprints, and other non-peer-reviewed publications; (2) studies involving participants younger than 60 years, studies with mixed-age samples from which data on older adults could not be separately extracted, and studies that provided insufficient information to confirm that the participants were older adults; (3) studies that did not involve therapeutic landscapes or health-promoting landscapes related to nature-based interventions or healing approaches; (4) studies that addressed only physical health, physical functioning, or social health outcomes without examining mental health; and (5) studies for which the full text was unavailable, key information was incomplete, or the publication was duplicated.

### 2.3 Study selection

**Figure 1:**
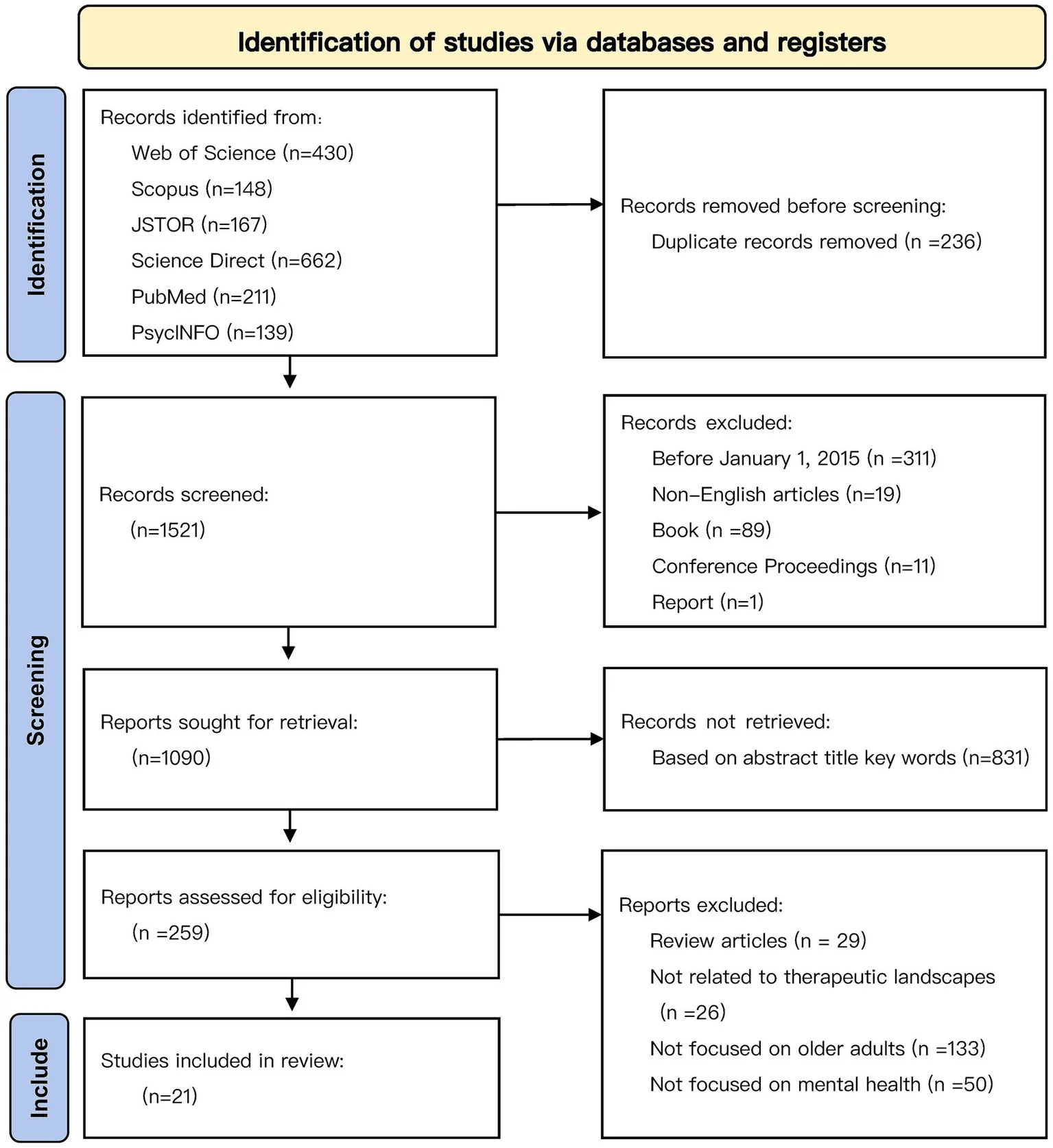
PRISMA flow diagram (Page et al., 2021).

This systematic review followed the PRISMA guidelines to ensure a systematic and transparent literature search and study selection process. A total of 1,757 records were identified through searches of six databases. After 236 duplicate records were removed, 1,521 records remained for further screening. The records were first screened according to publication type and basic eligibility criteria. This process excluded 311 records published outside the predefined time range, 19 non-English records, 89 books, 11 conference papers, and one report, leaving 1,090 records. The titles and abstracts of the remaining records were then screened, and 831 records unrelated to the topic of this systematic review were excluded. The full texts of the remaining records were subsequently assessed for eligibility according to the PICOS framework. At this stage, 29 review articles or non-empirical studies, 26 studies that did not involve therapeutic landscapes, 133 studies that did not include older adults as the study population, and 50 studies that did not assess mental health outcomes were excluded. Ultimately, 21 studies were included in the systematic review (Figure 1). Study selection was conducted independently by two reviewers. When disagreements arose, a third expert participated in the discussion until consensus was reached.

### 2.4 Methodological quality and risk of bias assessment

This study used the Crowe Critical Appraisal Tool (CCAT) to assess the methodological quality of the studies included in this systematic review (Crowe and Sheppard, 2011). The CCAT is a general critical appraisal tool applicable to quantitative, qualitative, and mixed-methods studies. It consists of eight appraisal domains: research background and objectives (Preliminaries/Introduction), research design (Design), sampling and sample characteristics (Sampling), data collection methods (Data Collection), data analysis procedures (Data Analysis), ethical considerations (Ethics), presentation of results (Results), and conclusions and research implications (Discussion and Conclusions) (Crowe, 2013). Each domain was scored on a scale of 0 to 5, yielding a total score ranging from 0 to 40. Studies with a total score of ≥30 were classified as having high methodological quality, those scoring 22–29 were classified as having moderate methodological quality, and those scoring below 22 were classified as having low methodological quality. Studies assessed as being of low quality were excluded from this systematic review (Crowe et al., 2012).

The results showed that all 21 included studies scored ≥30. Among them, 16 studies scored between 36 and 40, and 5 studies scored between 30 and 35, indicating that all included studies met the criteria for high methodological quality. Overall, the included studies demonstrated good methodological quality and provided a relatively reliable evidence base for the synthesis and analysis conducted in this systematic review (Table 3). Nevertheless, some of the included studies had methodological limitations. Specifically, two studies provided insufficient information on ethical review and approval (Lu et al., 2021; Zhang et al., 2021). Because these studies involved data collection methods such as questionnaires, interviews, and observations, the absence of adequate ethical review information may reduce the credibility of their findings and the transparency of their research procedures. Two studies also showed weaknesses in sampling, including insufficient justification for and limited representativeness of the cases selected for the analysis of therapeutic landscapes, as well as limited samples of older adults and institutions. These limitations may restrict the robustness and generalizability of the findings (Zaťovičová and Kubánová, 2024; Akindejoye et al., 2025). In addition, although two studies explained their main findings and discussed their limitations and directions for future research, their consideration of potential research bias, the generalizability of the findings, and causal relationships was insufficient (Zhang et al., 2021; Dempsey et al., 2018). Although most of the included studies employed cross-sectional or qualitative designs, which limited their ability to establish causal relationships between variables, they generally performed well in terms of the formulation of research questions, theoretical frameworks, data collection, reporting of results, and discussion. Collectively, these studies provide a relatively solid evidence base for understanding the relationship between therapeutic landscapes and the mental health of older adults and offer clear directions for future longitudinal and intervention studies with more rigorous research designs.

**Table 3:** Quality of studies assessed using the Crowe critical appraisal tool (CCAT).

Items	P	I	DE	S	DC	EM	R	DI	T
Dempsey et al. (2018)	5	5	4	4	5	5	4	3	35
Sia et al. (2018)	5	5	5	5	5	5	4	4	38
Samadi-Todar and Hami (2025)	5	4	4	5	4	5	5	4	36
Lin et al. (2025)	5	5	5	5	5	4	5	5	39
Lu et al. (2021)	4	5	5	4	4	3	5	4	34
Hao et al. (2024)	5	5	5	5	5	4	5	5	39
Zhang et al. (2021)	4	5	5	5	4	3	4	3	33
Zumelzu et al. (2025)	5	5	5	5	5	5	5	5	40
Hami et al. (2025)	5	5	4	5	5	5	4	5	38
Rose and Lonsdale (2016)	5	5	5	5	5	4	5	4	38
Milligan et al. (2015)	5	5	5	5	5	4	5	5	39
Zaťovičová and Kubánová (2024)	5	4	3	3	3	4	4	4	30
Cai et al. (2023)	5	4	5	4	4	5	5	4	36
Finlay et al. (2015)	5	5	5	5	5	5	5	4	39
Browning et al. (2019)	5	5	5	5	5	4	4	5	38
Finlay (2018)	5	4	5	5	5	5	5	4	38
Milligan et al. (2021)	5	4	5	4	5	5	5	4	37
Akindejoye et al. (2025)	4	5	4	3	4	5	4	5	34
van Houwelingen-Snippe et al. (2023)	5	5	5	4	5	5	5	5	39
Guo et al. (2024)	5	5	5	5	5	5	5	5	40
Corley et al. (2021)	5	5	4	4	5	5	5	5	38

P, preliminaries; I, introduction; DE, design; S, sampling; DC, data collection; EM, ethical matters; R, results; DI, discussion; T, total.

This study also used the JBI Critical Appraisal Tools developed by the Joanna Briggs Institute (Joanna Briggs Institute, 2017) to assess the risk of bias in the included studies. Depending on the study design, the JBI critical appraisal checklists for Analytical Cross-Sectional Studies (Barker et al., 2026), Qualitative Research (Porritt et al., 2024), Randomized Controlled Trials (Barker et al., 2023), and Quasi-Experimental Studies were applied (Barker et al., 2024). The appraisal results for each item included Yes, No, Unclear, and N/A. A response of Yes was scored as 1 point, whereas No and Unclear were scored as 0 points. N/A was excluded from the denominator when calculating the percentage score. The criteria for determining the risk of bias were as follows: a score of ≥70% was classified as Low risk, 50%–69% as Moderate risk, and <50% as High risk. Studies classified as having a High risk of bias were excluded from this systematic review. Because the number of items varied across the different checklists, only the overall score and risk-of-bias category for each study were reported in the main text, while the item-level appraisal results were provided in Supplementary File S2. All methodological quality and risk-of-bias assessments were independently conducted and cross-checked by two researchers. When disagreements arose, a third researcher participated in the discussion, and consensus was reached.

The results of the risk-of-bias assessment are presented in Table 4. Overall, the included studies demonstrated good methodological quality. Most studies were judged to have a Low risk of bias, whereas only a small number were classified as having a Moderate risk of bias. Among the studies assessed using the checklist for Randomized Controlled Trials, Sia et al. (2018), Hao et al. (2024), and the quantitative experimental component of the study by van Houwelingen-Snippe et al. (2023) were classified as having a Moderate risk of bias. This classification was mainly attributable to the readily identifiable nature of the horticultural activities and digital nature scenarios. Participants were generally able to determine which type of intervention they had received, which may have generated expectancy effects and influenced the assessment of subjective outcomes. Among the studies assessed using the checklist for Analytical Cross-Sectional Studies, Samadi-Todar and Hami (2025) were also classified as having a Moderate risk of bias. The main reasons were the limited evidence supporting the validity of the instrument used to measure psychological well-being and the failure to identify or account for potential confounding factors, such as age, educational attainment, and income, that may influence the mental health of older adults. Among the studies assessed using the checklist for Quasi-Experimental Studies, Akindejoye et al. (2025) were likewise classified as having a Moderate risk of bias. This was primarily because the study did not include a control group and involved a limited number of measurements before and after the nature-based intervention. Consequently, the observed changes in mental health may have been influenced by factors other than the intervention itself. Although a small number of studies were judged to have a Moderate risk of bias, their overall study designs, data collection procedures, and statistical analyses remained reasonably sound and were therefore able to provide relevant evidence for this systematic review.

**Table 4:** Risk of bias assessment.

Study	JBI checklist	Score	Percent (%)	Risk
Dempsey et al. (2018)	Analytical cross-sectional studies	7/8	87.5	Low risk
Sia et al. (2018)	Randomized controlled trials	8/13	61.5	Moderate risk
Samadi-Todar and Hami (2025)	Analytical cross-sectional studies	5/8	62.5	Moderate risk
Lin et al. (2025)	Analytical cross-sectional studies	5/6	83.3	Low risk
Lu et al. (2021)	Analytical cross-sectional studies	6/6	100	Low risk
Hao et al. (2024)	Randomized controlled trials	8/13	61.5	Moderate risk
Zhang et al. (2021)	Analytical cross-sectional studies	7/8	87.5	Low risk
Zumelzu et al. (2025)	Qualitative research	8/10	80	Low risk
Hami et al. (2025)	Analytical cross-sectional studies	6/8	75	Low risk
Rose and Lonsdale (2016)	Qualitative research	7/10	70	Low risk
Milligan et al. (2015)	Qualitative research	8/10	80	Low risk
Zaťovičová and Kubáňová (2024)	Qualitative research	8/10	80	Low risk
Cai et al. (2023)	Qualitative research	8/10	80	Low risk
Finlay et al. (2015)	Qualitative research	8/10	80	Low risk
Browning et al. (2019)	Analytical cross-sectional studies	8/8	100	Low risk
Finlay (2018)	Qualitative research	8/10	80	Low risk
Milligan et al. (2021)	Qualitative research	9/10	90	Low risk
Akindejoye et al. (2025)	Quasi-experimental studies	4/7	57.1	Moderate risk
van Houwelingen-Snippe et al. (2023)	Qualitative research	7/10	70.0	Low risk
	Randomized controlled trials	7/13	53.8	Moderate risk
Guo et al. (2024)	Analytical cross-sectional studies	7/8	87.5	Low risk
Corley et al. (2021)	Analytical cross-sectional studies	6/7	85.7	Low risk

Taken together, the results of the JBI risk-of-bias assessment and the CCAT methodological quality assessment indicated that the included studies were generally of high quality and that their findings had a reasonable degree of credibility. Therefore, all included studies were retained for the final data extraction and analysis stage, and no studies were excluded on the basis of these assessments.

## 3 Results

### 3.1 General characteristics

#### 3.1.1 Publication sources and characteristics

**Figure 2:**
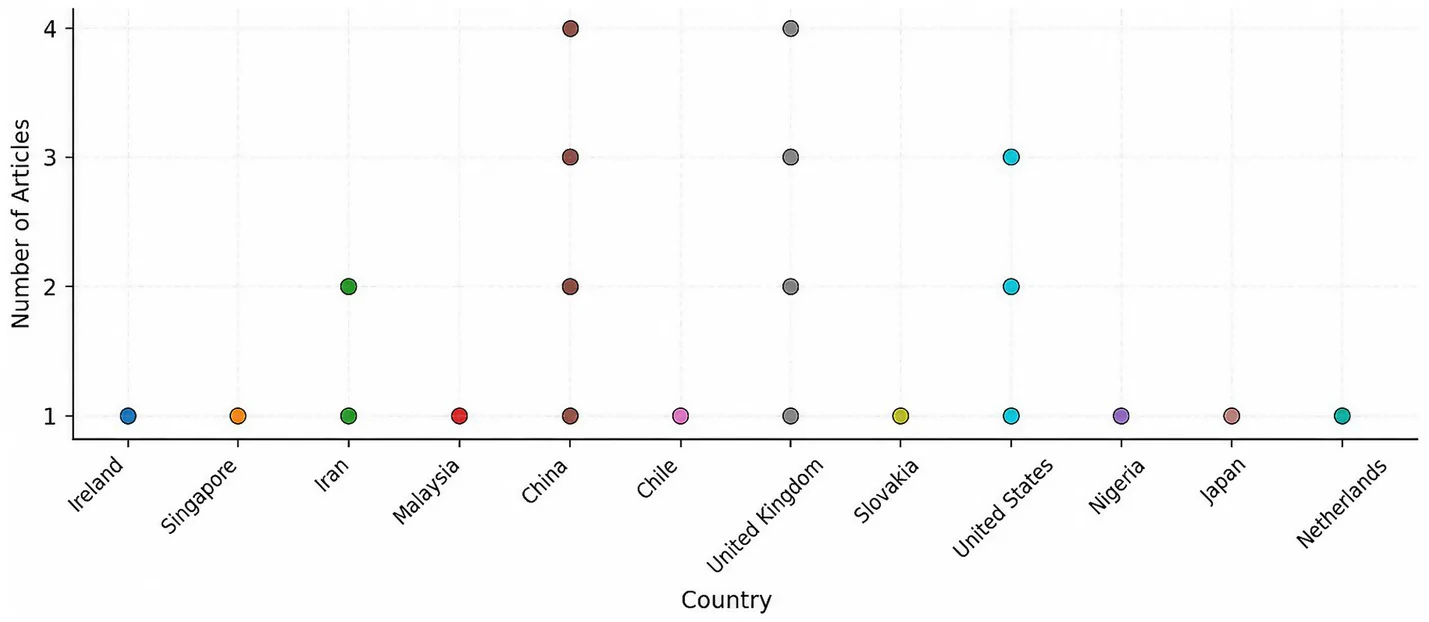
Geographical distribution of the 21 included studies by country.

The included empirical studies showed a certain degree of geographical diversity, mainly originating from Asia (*n* = 9), Europe (*n* = 7), and North America (*n* = 3), one study each from Chile in South America and Nigeria in Africa. In terms of country distribution, China (*n* = 4), the United Kingdom (*n* = 4), and the United States (*n* = 3) were the main contributors (Figure 2). The research content covered multiple types of natural therapeutic spaces, such as coastal spaces and wilderness environments, as well as urban therapeutic spaces, such as urban parks, built and neighborhood environments, outdoor spaces in older adult care institutions, and healthcare facilities. These studies also adopted various therapeutic intervention approaches, including horticultural therapy, art therapy, and physical exercise, and digital landscapes. Overall, this distribution indicates that current empirical research on therapeutic landscapes and the mental health of older adults is geographically concentrated, with Asia and Europe as the main centers of research. The studies are highly concentrated in a small number of countries, reflecting a clear imbalance in global research development. In particular, the research foundation remains relatively weak in developing countries and low-income regions.

**Figure 3:**
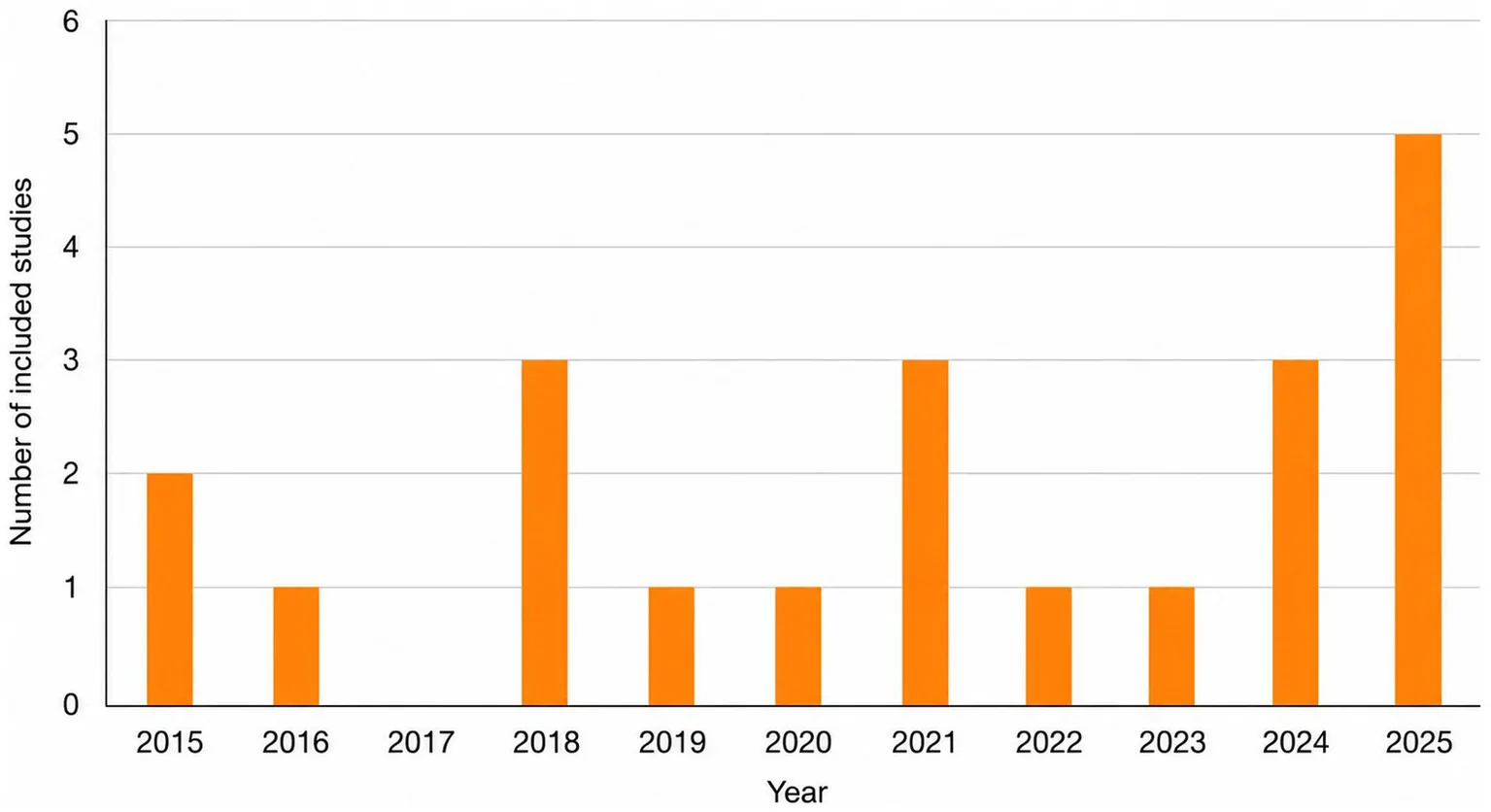
Annual number of included studies from 2015 to 2025.

In terms of annual publication volume, the number of included studies from 2015 to 2025 was generally low, but it showed a slow and fluctuating upward trend overall (Figure 3). The annual number of publications ranged from 0 to 5 studies. No studies meeting the inclusion criteria were identified in 2017, forming a clear gap in the publication timeline. After 2018, the annual number of publications fluctuated between 1 and 3 studies. Since 2023, the number of publications has increased year by year, reaching a temporary peak of 5 studies in 2025. Overall, this trajectory indicates that scholarly attention to the relationship between therapeutic landscapes and the mental health of older adults has fluctuated over time. Although scholarly attention has gradually increased in recent years, the overall scale of research remains limited and is still in the early stage of continued development.

In terms of journal distribution, the included studies were mainly published in journals within interdisciplinary fields. Among them, Health & Place (*n* = 4), Social Science & Medicine (*n* = 3), and Urban Forestry & Urban Greening (*n* = 3) published a relatively larger number of studies in this field (Table 5). These journals cover multiple disciplinary areas, including sociology, geography, medicine, environmental science, agricultural science, ecology, and engineering technology. Overall, existing studies are not concentrated in a single authoritative journal, but instead show a strong pattern of interdisciplinary publication. This reflects the significant comprehensiveness and interdisciplinarity of the topic of “therapeutic landscapes and the mental health of older adults”.

**Table 5:** Distribution of journals and years of publication.

Journal sources	2015	2016	2017	2018	2019	2020	2020	2022	2023	2024	2025	Totals
Ageing and Society	1	—	—	—	—	—	—	1	—	—	—	2
Emotion, Space and Society	—	—	—	—	—	—	—	—	1	—	—	1
Health & Place	1	1	—	1	—	—	—	—	—	—	1	4
Social Science & Medicine	—	—	—	1	—	—	2	—	—	—	—	3
Urban Forestry & Urban Greening	—	—	—	—	1	1	—	—	—	—	1	3
Journal of Therapeutic Horticulture	—	—	—	1	—	—	—	—	—	—	—	1
Hort Technology	—	—	—	—	—	—	—	—	—	1	—	1
Landscape and Ecological Engineering	—	—	—	—	—	—	—	—	—	—	1	1
Landscape Architecture and Sustainability	—	—	—	—	—	—	—	—	—	—	1	1
Slovak Journal of Civil Engineering	—	—	—	—	—	—	—	—	—	1	—	1
Occupational Therapy in Health Care	—	—	—	—	—	—	—	—	—	—	1	1
Journal of Environmental Psychology	—	—	—	—	—	—	1	—	—	1	—	2

#### 3.1.2 Participant characteristics

A total of 21 studies were included in this systematic review (Table 6), involving 6,071 participants in total. The sample sizes ranged from a minimum of 12 participants (Milligan et al., 2021) to a maximum of 3,688 participants (Dempsey et al., 2018). Only one study specifically focused on 52 older men (Milligan et al., 2015), one study was a design case study and did not include participants (Zaťovičová and Kubánová, 2024), and one study did not report the sex of the participants (Finlay et al., 2015). Among the included participants, women accounted for 52.89%, whereas men accounted for 47.11%. In addition, among the samples included in this systematic review, older adults aged 60 years or older accounted for 68.9%, whereas participants aged 49–60 years accounted for 31.1%.

**Table 6:** Characteristics of the studies.

Authors (Year)	TL type	Age	Population	MH Dimension	Measurement	Study designs	Outcome
Dempsey et al. (2018)	Coastal blue spaces	≥50 years	3,688 M 4351F	depressive symptoms	Depression scale (CES-D)	Cross-sectional survey	Sea-view ↑ → CES-D ↓; distance → NS (views stronger)
Sia et al. (2018)	Horticultural Therapy	60–85 years	13 M 46F	Psychological well-being	Ryff’s Scale of Psychological Well-Being	RCT, Crossover	Therapeutic horticulture → positive relations with others ↑
Samadi-Todar and Hami (2025)	Golestan Park	≥60 years	273 M	Mental well-being	5-point Likert scales	Cross-sectional survey	Physical activity ↑ → mental well-being ↑
Lin et al. (2025)	Community parks	≥60 years	43 M 32F	Physical; Social; Cultural	Community participation ability tool	Cross-sectional survey	Physical & cultural/symbolic qualities ↑ → mental well-being ↑
Lu et al. (2021)	Healing landscape plants	≥60 years	26 M 32F	Loneliness; Anxiety; happiness; depression	5-point Likert scales	AHP model	Physical activity ↑ → mental well-being↑
Hao et al. (2024)	Crape myrtle observation	60–99 years	43 M 47F	Positive affect; negative affect; overall mood	Smile face scale	RCT, Crossover	Flower viewing → positive affect ↑, negative affect ↓, overall mood ↑
Zhang et al. (2021)	Seaside health tourism destination	45–55 years	160 M 235F	Material; Social; Symbolic	Perceived health scale; General perceived health Scale	Cross-sectional survey	Social and symbolic landscapes↑ → better health perception↑
Zumelzu et al. (2025)	Neighborhood walking environment	≥60 years	25 M 25F	Mental wellbeing during walking	semi-structured interviews [30–50 min]	Qualitative go-along walking interviews	Walkable streets and greenery → mental wellbeing↑
Hami et al. (2025)	NHs	≥61 years	142 M 135F	Liveliness; sense of comfort	5-point Likert scales	Cross-sectional survey	Vegetation diversity and green views → mental well-being ↑
Rose and Lonsdale (2016)	re-imagining landscape	65–86 years	3 M 20F	Mood; Wellbeing; self-worth; social connectivity	group discussion; in-depth interviews	Qualitative case study	e-imagining landscape through painting ↑ → subjective well-being ↑
Milligan et al. (2015)	Men in Sheds	49–87 years	52 M	Loneliness; Social; isolation; mood	Semi-structured interviews; Focus group discussions [5–12 min]	Qualitative case study	Shed participation→ less loneliness and wellbeing↑
Zaťovičová and Kubáňová (2024)	Hospital healing garden	N/A	N/A	Stress; anxiety; dementia; memory	Field study	Case study	Healing garden→elderly mental well-being↑
Cai et al. (2023)	Urban park singingscape	≥60 years	11 M 13F	mental relaxation; social belonging; emotional imagination	In-depth interviews	Qualitative case study	Singing in parks → relaxation & wellbeing; belonging ↑
Finlay et al. (2015)	Blue & green spaces	≥65 years	141	psychological health; renewal; stress; spiritual connectedness	Semi-structured interviews [60–90 min] walking interviews [~15 min]	Qualitative case study	Blue/green spaces → psychological health ↑; blue spaces especially → relaxation & stress reduction
Browning et al. (2019)	NHs	≥65 years	9,186 NHs; female 70.32%	Depressive symptoms	Long-stay residents with depressive symptoms (%).	Cross-sectional survey	Tree canopy cover ↑ → depressive symptoms ↓
Finlay (2018)	White space	55–92 years	125	Mental well-being; Social well-being	Semi-structured interviews; participant observation	Qualitative case study	White space snow and ice → worry fear stress ↑; happiness identity social bonds ↑
Milligan et al. (2021)	Wilderness therapeutic landscape	52–75 years	5 M 7F	psychological and social aspects	In-depth interviews	Qualitative case study	Wilderness nature-adventure → well-being ↑
Akindejoye et al. (2025)	Co-designed healing gardens	60–99 years	14 M 19F	Quality of life; agitation; depression	DEMQOL-Proxy; CMAI; CSDD; GuOQ	Quasi-experimental pre-post study	Healing gardens → quality of life ↑ and agitation ↓; depression ↑ (no beneficial change)
van Houwelingen-Snippe et al. (2023)	Digital nature	Study 1: 69–92 years; Study 2: ≥55 years	Study 1: 6 M 20F; Study 2: 69 M 131F	Social aspirations; awe; fascination; sense of presence	Focus groups; Social Aspirations Scale; Awe Experience Scale; Perceived Restorativeness Scale; Spatial Presence Experience Scale	Mixed methods: focus groups and 2 × 2 mixed experiment	Tended nature → social aspirations, awe, fascination, and sense of presence ↑; dense scenes → fascination and presence ↑
Guo et al. (2024)	Community gardens in elderly housing with care services	65–96 years	145 M 177F	Positive well-being; self-rated health	WHO-5 Well-Being Index; self-rated health item	Cross-sectional survey	Garden visit frequency and perceived garden quality ↑ → positive well-being and self-rated health ↑; outing frequency and social cohesion mediated positive well-being
Corley et al. (2021)	Home gardens	84 ± 0.5 years	91 M 80F	Emotional and mental health; anxiety; sleep quality	Self-rated health items; composite well-being score	Cross-sectional survey	Garden use ↑ → emotional/mental health and sleep quality ↑; gardening or relaxing in the garden → NS

NHs, nursing homes; N/A, not applicable; TL, therapeutic landscape; M, male; F, female; N/A, not applicable; MH, mental health; RCT, Randomized Controlled Trial; AHP, analytic hierarchy process; NS, not significant; ↑, Increase; ↓, Decrease; →, Affects.

### 3.2 Measurement and intervention approaches

#### 3.2.1 Measurement tools

In the studies included in this systematic review, older adults’ mental health was primarily assessed using subjective rating scales, including, a depression scale (Dempsey et al., 2018), Ryff’s Psychological Well-Being Scale (Sia et al., 2018), a community participation ability scale (Lin et al., 2025), a Smiley Face Scale (Hao et al., 2024), the Dementia Quality of Life Proxy measure (DEMQOL-Proxy), the Cohen-Mansfield Agitation Inventory (CMAI), the Cornell Scale for Depression in Dementia (CSDD) (Akindejoye et al., 2025), the Social Aspirations Scale, the Awe Experience Scale, the Perceived Restorativeness Scale, the Spatial Presence Experience Scale (van Houwelingen-Snippe et al., 2023) a self-rated health scale, and a general self-rated health scale (Zhang et al., 2021). Samadi-Todar and Hami (2025), Lu et al. (2021), and Hami et al. (2025) used 5-point Likert-type scales, with scores ranging from 1 (strongly disagree) to 5 (strongly agree). Dempsey et al. (2018) used a depression scale to reflect the relationship between participants’ depressive mood and mental health and to assess the severity of depressive symptoms among older adults. Sia et al. (2018) used Ryff’s Psychological Well-Being Scale to measure the psychological well-being of older adults. Lin et al. (2025) used a community participation ability scale to assess older adults’ sense of social belonging and social well-being in relation to mental health. Hao et al. (2024) used a Smiley Face Scale to measure older adults’ emotional states, including positive and negative emotions. Zhang et al. (2021) used a self-rated health scale and a general self-rated health scale to measure older adults’ sense of psychological security, psychological comfort, and psychological well-being.

In addition to scale-based measurements, the studies included in this systematic review also assessed older adults’ mental health through semi-structured interviews, in-depth interviews, and focus group discussions (Zumelzu et al., 2025; Rose and Lonsdale, 2016; Milligan et al., 2015; Cai et al., 2023; Finlay et al., 2015; Finlay, 2018; Milligan et al., 2021). Zumelzu et al. (2025), Milligan et al. (2015), Finlay et al. (2015), and Finlay (2018) measured older adults’ loneliness, emotional states, stress levels, and psychological well-being through semi-structured interviews and focus group discussions. Cai et al. (2023) and Milligan et al. (2021) examined older adults’ subjective experiences of mental health through in-depth interviews, including emotional states and relaxation, loneliness and social connectedness and stress and emotional regulation. In addition, only one design case study focusing on hospital gardens and nursing home gardens examined the potential mental health benefits of green environments for older adults’ mental health in landscape design practice (Zaťovičová and Kubánová, 2024).

#### 3.2.2 Therapeutic landscape intervention approaches

The types of therapeutic landscape interventions covered by the studies included in this systematic review mainly nature-based landscapes characterized by green spaces, blue spaces, and white spaces; structured intervention landscapes represented by horticultural therapy, plant viewing, and wilderness experiences, and digital nature; and sociocultural experiential landscapes formed through group-based cultural activities in parks, such as choir singing, landscape painting, and workshops. The effects of these different landscape interventions on the mental health of older adults varied to some extent.

Within nature-based landscapes, studies of blue spaces, primarily coastal environments, and green spaces, including urban parks, community green spaces, greenery in residential care facilities for older adults, community gardens, and home gardens, indicated that greater exposure to natural environments was associated with fewer depressive symptoms, higher psychological well-being, better self-rated health, and lower stress levels. For example, Dempsey et al. (2018) found that exposure to blue spaces as an intervention was associated with a reduced risk of depression. Finlay et al. (2015) reported that interventions involving blue and green spaces improved the social health and quality of life of older adults. Zhang et al. (2021) examined the influence of natural, social, and symbolic landscapes on perceived health. However, winter-dominated white landscapes exhibit a dual health effect. Winter outdoor activities such as skiing and skating can increase older adults’ opportunities to engage with nature and enhance their sense of well-being. However, low temperatures, snow accumulation, and icy surfaces may reduce environmental safety and accessibility, thereby increasing concerns about falls and injuries (Finlay, 2018). Therefore, the actual effects of winter landscape interventions depend on the balance between their capacity to promote activity and the environmental safety risks they entail.

Compared with studies of exposure to natural environments, research on structured intervention landscapes included a greater number of experimental studies and pre–post intervention studies. For example, Sia et al. (2018) found that horticultural therapy interventions improved psychological well-being. Lu et al. (2021) used plant landscape interventions to promote the psychological and physiological rehabilitation of older adults. Hao et al. (2024) supported this view and reported that participation in crape myrtle viewing activities helped improve older adults’ physiological indicators and emotional states. Zumelzu et al. (2025) examined how features of the built environment, such as walkability and access to shops, influenced residents’ well-being in later life. Van Houwelingen-Snippe et al. (2023), meanwhile, used virtual reality technology to investigate older adults’ preferences for digital nature and suggested that digital nature had positive potential to enhance social well-being and improve emotional states. Milligan et al. (2021) used “wilderness” therapeutic landscapes as an intervention, focusing primarily on older adults’ behaviors, and emphasized that participation in adventure activities was beneficial to their physical and mental health. Overall, structured intervention landscapes may improve older adults’ emotional states, psychological well-being, and social connectedness through organized contact with nature, activity participation, and social interaction. However, the existing evidence mainly supports short-term effects, while the long-term sustainability and general applicability of these effects require further verification through high-quality longitudinal and controlled studies.

Sociocultural experiential landscapes are largely grounded in social activities and humanistic care. Through emotional arousal, social participation, and a sense of group belonging, they may alleviate anxiety, stress, and loneliness among older adults and enhance their psychological well-being. For example, Rose and Lonsdale (2016) used painting as an intervention to improve older adults’ subjective well-being by evoking their feelings and memories. Milligan et al. (2015) reported that the transformation of a “Men’s Shed” helped improve the physical and mental health of older men, maintain their physical and psychological well-being, and reduce social isolation. Cai et al. (2023) used a “singingscape” as an intervention to promote the physical and mental health of older adults and strengthen their sense of collective belonging. However, the existing evidence is derived primarily from qualitative studies, and the magnitude of the effects of sociocultural experiential landscapes, as well as the independent contribution of the environment itself, requires further investigation.

### 3.3 Effects of therapeutic landscapes on older adults’ mental health

A total of 21 studies were included in this systematic review. The results showed that therapeutic landscapes can influence the mental health of older adults by providing restorative experiences and increasing contact with nature and opportunities for social interaction (Zhang et al., 2025). The effects of therapeutic landscapes on older adults’ mental health are multidimensional and are mainly manifested in depression, anxiety, stress, loneliness, and psychological well-being.

#### 3.3.1 Depression

Depression is one of the relatively common mental health problems among older adults (Das et al., 2025b). Most studies have shown that exposure to natural environments has positive effects on alleviating depressive symptoms and improving overall emotional states. For example, Dempsey et al. (2018) found that visual exposure to blue spaces may promote mental health. In particular, exposure to coastal blue spaces was significantly associated with a lower incidence of depression. This indicates that, in residential planning and design, attention should be paid to residential orientation, window placement, and overall landscape view corridors to ensure that older adults can obtain stable and continuous visual exposure to blue spaces in their daily lives. Browning et al. (2019) examined the effects of green space coverage in nursing homes on depressive symptoms among older adults. The results showed that as green space coverage increased, older adults’ depressive symptoms were significantly alleviated, thereby improving their overall mental health status. This measure was regarded as a cost-effective and scalable environmental intervention approach. However, a small number of studies were unable to establish a causal relationship between exposure to natural landscapes and depressive symptoms. For example, the quasi-experimental study by Akindejoye et al. (2025) found that although agitation among older adults decreased following their use of the healing garden, their depression scores did not improve. Therefore, the current evidence provides stronger support for a possible association between therapeutic landscapes and fewer depressive symptoms, but remains insufficient to demonstrate a stable antidepressant effect.

#### 3.3.2 Anxiety

Anxiety is one of the most easily overlooked mental health problems among older adults and is often accompanied by a range of psychological, behavioral, and neurological symptoms. For example, older adults may experience communication difficulties, depression, dementia, and neurological decline, which may further increase the risk of suicide (Bryant et al., 2008). The positive effects of therapeutic landscapes on anxiety among older adults have been supported by most of the included studies. During the COVID-19 pandemic, most older adults who had access to home gardens reported no anxiety or lower levels of anxiety, even when they were confined to their homes (Corley et al., 2021). Furthermore, even without direct contact with nature, viewing videos showing plants in bloom could significantly alleviate negative emotions, reduce tension and anxiety among older adults, and thereby promote emotional recovery and improve mental health. Hao et al. (2024) demonstrated these effects through an experiment involving videos of blooming crape myrtles. Sia et al. (2018), through a randomized controlled trial, found that horticultural therapy could significantly promote mental health among multiethnic older adults in Singapore, particularly by alleviating attention fatigue and reducing anxiety, while also decreasing anxiety-induced feelings of insecurity and loneliness. Cai et al. (2023) examined the “singingscape” jointly created through singing activities, the spatial environment, participant interactions, and emotional experiences. Their findings suggest that the singingscape facilitated communication among older adults, promoted feelings of calmness and relaxation through social interaction, alleviated anxiety-related loneliness and depressive emotions, and potentially reduce the risk of suicide.

#### 3.3.3 Stress

Stress symptoms have negative effects on older adults’ mental health and well-being. For example, they are closely associated with mental health problems, difficulty falling asleep, low mood, and somatic symptoms (Ebrahimi et al., 2022). Samadi-Todar and Hami (2025) indicated that urban parks and green space coverage have important effects on the mental health of older men. Increases in park green space area and tree canopy coverage can effectively alleviate stress-related distress among older adults, for example, by reducing environmental noise through increased vegetation coverage. Meanwhile, improving the comfort of park seating, shelters, and recreational areas can prolong the time older adults spend in parks and increase opportunities for social interaction, thereby reducing their psychological stress levels. Hami et al. (2025) further supported this view, finding that urban green spaces can alleviate stress, depression, and anxiety among older adults. Although green space environments can reduce older adults’ exposure to environmental stressors—for example, through vegetation that buffers noise and dust—and thereby lower their stress levels, evidence regarding the stress-reducing effects of some natural environments remains inconsistent. For example, Finlay (2018) found that snowy and icy environments may increase worry and psychological stress because of cold conditions, slippery surfaces, and an increased risk of falls. Therefore, the stress-reducing effects of natural environments on older adults are not universal but depend on climatic conditions, environmental safety, and older adults’ physical functional status.

#### 3.3.4 Loneliness

With increasing age, older adults tend to experience loneliness and face social isolation (Kemperman et al., 2019). Lin et al. (2025) indicated that community parks, as a type of therapeutic landscape, provide older adults with spaces for social interaction and opportunities for social engagement. Features such as continuous and level walkways, street-facing shops and community service points, street greenery, well-maintained front gardens, and abundant trees and lawns can significantly enhance older adults’ sense of tranquility, safety, and trust in public spaces during daily walking. These features also promote interaction with neighbors and experiences of community places, thereby strengthening social connections and reducing feelings of loneliness. Lu et al. (2021) highlighted that horticultural therapy has a significant effect on promoting older adults’ physical and mental health. They emphasized that plants are not merely background greenery, but serve as core mediators of restorative environmental perception. In addition to providing positive stimulation through visual, olfactory, and tactile experiences, when environmental perception mediated by plants is linked to older adults’ life memories, it can enhance the psychological restorative potential of the environment and foster place attachment, thereby reducing loneliness and alleviating tension among older adults.

#### 3.3.5 Psychological well-being

Mental health is not limited to whether an individual has been diagnosed with a mental disorder; it also encompasses the emotional experiences, life satisfaction, and overall self-evaluation that an individual demonstrates over a given period. Samadi-Todar and Hami (2025) found that aesthetically pleasing and well-equipped park environments, as well as sufficient physical activity undertaken within them, were significantly and positively associated with the psychological well-being of older adults. Hami et al. (2025) showed that nursing homes and outdoor courtyards with greater levels of nature exposure were more likely to enhance older adults’ environmental satisfaction, psychological well-being, and social interaction. Guo et al. (2024) similarly found that the frequency of visits to community gardens and perceived garden quality were associated with positive well-being and self-rated health, and that the frequency of going outdoors and neighborhood social cohesion might play mediating roles. Corley et al. (2021) reported that more frequent garden use was associated with better mood and mental health. Meanwhile, Milligan et al. (2015) indicated that informal, low-barrier, and practice-oriented community activity spaces could also provide older adults with a sense of purpose, belonging, and peer support. Taken together, these findings suggest that urban parks, nursing home environments, and community spaces characterized by greater nature exposure, convenient accessibility, and age-friendly facilities, together with various practice-oriented activity programs, may jointly promote subjective well-being among older adults.

In addition, psychological well-being is also reflected in the activities undertaken within landscape spaces. In terms of therapeutic activities, the included studies mainly focused on horticultural therapy, outdoor adventure, community choir singing, and painting practice. For example, Sia et al. (2018) found that sustained horticultural activities could not only provide older adults with healthy leisure opportunities but also enhance their subjective well-being. Milligan et al. (2021) argued that the challenges and risks encountered in wilderness environments could enhance older adults’ sense of personal accomplishment, self-efficacy, and social engagement, thereby reducing stereotypes associated with aging, alleviating psychological stress, and improving subjective well-being and self-perceived vitality. Rose and Lonsdale (2016) found that the process of “landscape painting” could reconstruct the emotional connections between older adults and places, thereby enhancing their subjective well-being and emotional states. These findings indicate that the therapeutic effects of therapeutic landscapes are not determined solely by the physical attributes of space. Rather, their integration with multidimensional therapeutic activities can more effectively stimulate older adults’ social participation and sensory experiences. The planning and design of therapeutic landscapes should therefore consider spatial environments and therapeutic activities in an integrated manner to create a comprehensive therapeutic system in which they mutually support each other. Meanwhile, age-friendly design should be guided by older adults’ subjective experiences and jointly shaped by spatial elements characterized by high accessibility, abundant natural vegetation, a strong sense of place, and distinctive cultural imagery.

## 4 Discussion

### 4.1 General characteristics of the included studies

In terms of study sources and publication characteristics, the geographical distribution of the included studies was uneven. Studies conducted in China placed greater emphasis on translating research findings into age-friendly landscape design strategies and more commonly employed quantitative methods (Lu et al., 2021; Zhang et al., 2021; Hao et al., 2024; Lin et al., 2025; Cai et al., 2023). Studies from the United Kingdom focused more on older adults’ subjective experiences and social relationships and more commonly adopted qualitative methods (Rose and Lonsdale, 2016; Milligan et al., 2015; Milligan et al., 2021; Corley et al., 2021). Research from the United States was more diverse in focus and placed greater emphasis on the relationships among levels of nature exposure, environmental risks, and psychological outcomes (Browning et al., 2019; Finlay, 2018).

In terms of the disciplinary distribution of publications, the included studies were not confined to a single issue within landscape studies or environmental design but received attention from multiple disciplines. This interdisciplinary publication pattern reflects the inherently integrative nature of this research topic. On the one hand, therapeutic landscapes involve the physical characteristics of spatial environments and related design interventions (Williams, 1998). On the other hand, older adults’ mental health is closely associated with complex factors such as social support, physical functioning, emotional restoration, and health inequalities (Gesler, 1992). Therefore, research in this field naturally requires the integration of multidisciplinary theories and methods. Meanwhile, the existing literature has not yet formed a relatively concentrated core publication platform, which, to some extent, suggests that the disciplinary boundaries of this topic are still evolving. Its theoretical frameworks and methodological paradigms also require further unification and development.

In terms of participant characteristics, the relatively high proportion of female participants in the included studies may be related to older women’s greater willingness to participate in research on health promotion, community engagement, and caregiving, as well as the higher proportion of women among the oldest-old population (Lee and Yeung, 2019; Arber and Cooper, 1999). However, only a small number of current studies have specifically focused on older men, and some studies did not even report participants’ gender information, indicating that the gender dimension has not yet received sufficient attention (Milligan et al., 2015). In terms of age structure, existing studies have mainly focused on older adults aged 60 years or older, while insufficient attention has been paid to individuals aged 49–60 years in the pre-old-age or transition-to-old-age stage (Carver et al., 2024). Future research should strengthen gender- and age-stratified analyses and further compare the differential effects of therapeutic landscapes on the mental health of different groups of older adults, thereby improving the specificity and practical guidance value of the research findings.

In terms of research designs and measurement instruments, most of the 21 included studies adopted cross-sectional designs, while a smaller number used randomized controlled trials, quasi-experimental designs, qualitative methods, or mixed-methods approaches. Cross-sectional studies can collect data from relatively large samples to examine associations between landscape exposure and mental health. However, because data are collected at a single point in time, these studies can only demonstrate associations and cannot establish causal relationships (Setia, 2016). In addition, most studies relied on questionnaires and self-report scales to assess mental health. Although these instruments are convenient to administer and relatively inexpensive, their results depend heavily on participants’ subjective reports and are susceptible to recall bias, social desirability effects, momentary emotional states, and differences in comprehension ability (Althubaiti, 2016). Some studies also used researcher-developed items, single-item indicators, or caregiver-proxy assessments, which may have lower validity than standardized scales. Furthermore, the instruments used varied across studies, with differences in measurement dimensions, scoring methods, and classification criteria, thereby limiting the comparability and generalizability of the findings. By comparison, randomized controlled trials can effectively reduce between-group baseline differences and the influence of confounding factors through random allocation and the establishment of control conditions, thereby providing a relatively stronger basis for causal inference (Hariton and Locascio, 2018). However, only a limited number of the included studies adopted this design, and these studies commonly had small sample sizes, short intervention periods, and outcome measures that continued to rely primarily on subjective scales (Sia et al., 2018; Hao et al., 2024). Quasi-experimental and online experimental studies can compare psychological changes before and after an intervention or under different landscape conditions (Akindejoye et al., 2025; van Houwelingen-Snippe et al., 2023). Qualitative interviews, focus groups, and participant observation can provide an in-depth understanding of older adults’ lived experiences and the specific processes through which therapeutic landscapes exert their effects (Milligan et al., 2015; Cai et al., 2023; Finlay et al., 2015; Malterud, 2001). The analytic hierarchy process and site-based case analyses can further translate research findings into landscape evaluation indicators and design recommendations (Lu et al., 2021; Zaťovičová and Kubánová, 2024). Nevertheless, these studies generally had relatively small samples, limited participant representativeness, a heavy reliance on subjective assessments, and substantial variation in research contexts. Overall, different research methods offer distinct advantages in identifying associations, supporting causal inference, interpreting subjective experiences, and translating findings into design practice. However, differences in research designs, measurement instruments, and evaluation criteria reduce the comparability of findings across studies. Future research should strengthen multicenter randomized controlled trials, longitudinal follow-up studies, and mixed-methods designs. It should also integrate standardized psychological scales with objective and real-time measurement tools, including wearable sensors, ecological momentary assessment, and GPS trajectories, to improve the accuracy and temporal sensitivity of measurements of landscape exposure and psychological responses (Shiffman et al., 2008).

### 4.2 Overall effects of therapeutic landscapes on older adults’ mental health

A total of 21 studies were included in this systematic review. The findings indicate that therapeutic landscapes have positive effects on multiple mental health outcomes among older adults, including anxiety, depression, stress, loneliness, and well-being. Zaťovičová and Kubánová (2024) synthesized the existing evidence and proposed a garden renovation plan, arguing that high-quality greenery could psychologically alleviate stress and anxiety and improve cognitive functioning and attention. These benefits could, in turn, enhance mobility, reduce the risk of falls and the need for medication, improve the overall quality of life of older patients, and potentially contribute to greater longevity. Finlay et al. (2015) suggested that the blue and green spaces encountered by older adults in their daily lives could foster psychological connections with place and provide opportunities to escape from everyday stress, thereby promoting psychological restoration. At the social level, these spaces could also facilitate communication and interaction among neighbors, family members, and friends, while strengthening older adults’ sense of self-worth, identity, and belonging (Finlay et al., 2015).

Although therapeutic landscapes have multiple positive effects on older adults’ mental health, one of the included studies also reported negative effects on older adults’ mental health. When environmental conditions exceed older adults’ physical adaptive capacity or adequate safety measures are lacking, natural environments with restorative potential may become new sources of stress. For example, Finlay (2018) noted that severe cold, icy conditions, and fall risks in white spaces may increase the likelihood of older adults avoiding outdoor activities and experiencing social isolation. This study broadens the overall understanding of the health effects of therapeutic landscapes and indicates that their effects do not always follow a uniformly positive direction. In fact, whether a landscape becomes a factor that promotes the mental health of older adults depends on the degree of fit between specific environmental characteristics and individual circumstances (Finlay et al., 2015). When landscape spaces have limited accessibility, high safety risks, uncomfortable climatic conditions, or do not correspond to older adults’ physical abilities and use requirements, their mental health benefits may be weakened or may even be transformed into sources of stress and experiences of exclusion (Finlay, 2018; Ottoni et al., 2016). Therefore, the therapeutic quality of a landscape should be understood as a contextual and relational attribute rather than as an inherent and universally valid characteristic of the landscape itself (Bell et al., 2018).

Future research should pay greater attention to unintended effects that therapeutic landscapes may produce, including anxiety, risk perception, activity avoidance, and social isolation. It should also compare differences across landscape types and groups of older adults and examine the moderating roles of climatic conditions, environmental safety, physical functioning, and individual characteristics. This would help clarify the pathways and boundary conditions through which landscape exposure, use, and experience influence mental health and improve the safety, precision, and applicability of therapeutic landscape design and interventions.

### 4.3 Differential effects of therapeutic landscape types on older adults’ mental health

The effects of different types of therapeutic landscapes on older adults’ mental health vary to some extent. Their health-promoting effects do not occur uniformly but are closely related to the spatial attributes of the landscapes, intervention approaches, and sociocultural meanings (Mossabir et al., 2021). Based on a synthesis of the included studies, this systematic review classified therapeutic landscapes into three types: nature-based landscapes, structured intervention landscapes, and sociocultural experiential landscapes. It further examined the differential effects of these therapeutic landscape types on older adults’ mental health.

#### 4.3.1 Nature-based landscapes

Nature-based landscapes constitute the foundation of therapeutic landscapes and have relatively widespread positive effects on older adults’ mental health. The natural landscapes examined in the included studies were not limited to a single type. Based on their environmental characteristics, nature-based landscapes could be classified into green spaces, blue spaces, and white spaces. Although these three types differ in their landscape forms and modes of experience, all have positive effects on emotional restoration, stress alleviation, the reduction of loneliness, and the enhancement of subjective well-being.

Among the studies included in this systematic review, green spaces were the most common type of therapeutic landscape, mainly including urban parks, community parks, home gardens, green spaces associated with older adult care facilities, hospital healing gardens, and street greenery. Existing studies showed that higher-quality green spaces and more frequent use were associated with greater psychological well-being, comfort, and perceived safety among older adults (Samadi-Todar and Hami, 2025; Zumelzu et al., 2025; Guo et al., 2024; Foettinger et al., 2022). Corley et al. (2021) further found that more frequent garden use was closely associated with better mental health and sleep quality. In addition, greater tree canopy cover was associated with fewer depressive symptoms, whereas greater vegetation diversity and a higher green view index were associated with stronger feelings of vitality and comfort (Browning et al., 2019; Hami et al., 2025). These findings indicate that the health effects of green spaces are jointly influenced by landscape quality, older adults’ perceptual preferences, actual frequency of use, and patterns of activity. In future design, whether green landscapes can provide older adults with safe, convenient, and sustainable opportunities for daily contact will be a key factor determining their effects on older adults’ mental health (Zumelzu et al., 2025).

Compared with green spaces, blue spaces tend to exhibit more pronounced advantages in emotional regulation and psychological relaxation. Their unique water environments are characterized by a high degree of openness, fluidity, and visual continuity, which can provide older adults with a stronger sense of calmness and psychological stability (Finlay et al., 2015; White et al., 2010). Finlay et al. (2015) found that blue and green spaces contributed to relaxation, psychological restoration, stress alleviation, and a sense of psychological connectedness. Zhang et al. (2021) also indicated that coastal health tourism landscapes were associated with restorative experiences and better perceived health. Dempsey et al. (2018) emphasized that both physical and visual proximity to blue spaces could provide low-stress environments and relatively gentle sensory stimulation, which were more conducive to maintaining psychological balance and reducing the risk of depression. However, the distance between participants’ residences and the coast was not significantly associated with depression. These findings suggest that the visibility, accessibility, landscape quality, actual frequency of use, and individual perception of blue spaces may be more influential than the residential distance between blue spaces and older adults’ homes. Future design should therefore place greater emphasis on the visibility, accessible distance, frequency of use, and patterns of activity associated with blue spaces to enhance their effectiveness as psychological interventions for older adults. Related research should also expand the geographical and population coverage of study samples, compare the differential effects of different types of blue spaces, and further examine potential mechanisms involving psychological restoration, physical activity, and social interaction.

White spaces with pronounced seasonal characteristics further demonstrate the context-dependent nature of the health effects of natural environments (Li et al., 2023). The purity, serenity, and seasonally novel experiences provided by snow- and ice-covered landscapes may, to some extent, promote visual relaxation and psychological comfort (Bao et al., 2023). However, factors such as cold weather, icy surfaces, the risk of slipping and falling, and restricted mobility may also diminish or even reverse their potential restorative effects (Curl et al., 2020). Finlay (2018) suggested that, for older adults, white spaces often represent not only aesthetic experiences of nature but also heightened environmental stress and risk perception. Therefore, research on white spaces indicates that natural environments are not inherently therapeutic; their health effects depend on the degree of fit between environmental conditions and individual capacities. Future white-space design should therefore preserve the aesthetic and restorative value of snow- and ice-covered landscapes while improving their safety and accessibility through measures such as snow removal and slip prevention, accessible routes, adequate lighting, and sheltered resting facilities. Future research should also compare differences in older adults’ experiences across climatic conditions, snow and ice conditions, and population groups. Longitudinal studies spanning multiple seasons are needed to assess the changing relationship between the psychological benefits and environmental risks of white spaces over time.

#### 4.3.2 Structured intervention landscapes

Structured intervention landscapes mainly involve active forms of interaction, such as horticultural therapy, plant viewing, wilderness experiences, and digital landscapes. By guiding older adults to establish sustained contact with natural environments, these interventions can enhance the specificity and practical applicability of psychological restoration. Compared with general exposure to natural environments, these interventions have clearly defined activity content and implementation processes and can increase older adults’ active participation through planting, viewing, physical activity, or digital experiences.

Horticultural therapy, as the most typical form of structured intervention landscape, produces positive effects not only through the restorative experience associated with contact with plants, but more importantly through the continuous process of planting, observing, and caring for them. Sia et al. (2018) found that horticultural therapy helped enhance older adults’ sense of participation, sense of control, and connections with others, while alleviating loneliness, feelings of meaninglessness, and negative emotions to some extent. Its particular advantage lies in helping older adults re-establish positive connections with the environment, time, the self, and society. In addition to caring for plants, Lu et al. (2021) and Hao et al. (2024) showed that simply observing and appreciating plants could also produce positive effects through passive viewing. Plants characterized by rich colors, fragrant scents, distinctive tactile qualities, and cultural symbolism are more likely to evoke feelings of pleasure and familiarity, memory associations, and emotional calmness among older adults (Song et al., 2018). Meanwhile, plant growth and changes, the flowering process, leaf forms, and fine textures may promote older adults’ immersive experiences and emotional regulation through sustained visual attention and the perception of vitality (Jeong et al., 2021; Mochizuki-Kawai et al., 2020). Structured nature-based interventions are not limited to horticultural therapy and plant viewing but can also extend from small-scale plant experiences to nature-adventure activities in wilderness environments. Milligan et al. (2021) found that activities such as hiking, mountain climbing, and cycling in wilderness environments could promote older adults’ psychological and social well-being through contact with nature, physical challenges, and social interaction. Compared with ordinary urban green spaces, the uncertainty and challenges associated with wilderness environments may further strengthen older adults’ sense of autonomy, self-efficacy, and meaning in life. In addition, digital landscapes can provide an alternative experience for older adults with limited mobility, those living in older adult care facilities, or those who have difficulty accessing real natural environments. Van Houwelingen-Snippe et al. (2023) found that digital nature could similarly evoke positive emotions, willingness to engage socially, feelings of awe, and restorative experiences. Among these digital environments, managed natural scenes that conveyed a sense of safety were more strongly preferred by older adults.

Overall, horticultural therapy and wilderness experiences can effectively integrate contact with nature, task completion, physical activity, and social interaction, thereby enhancing older adults’ sense of participation, sense of control, and self-efficacy. However, these interventions often require long-term maintenance and may be limited by inadequate safety and poor accessibility. By comparison, plant viewing and digital landscapes place fewer physical demands on participants and can provide alternative nature experiences for older adults with limited mobility. Nevertheless, they also have limitations, including limited activity formats, relatively short-lived therapeutic effects, and comparatively less immersive experiences. Future design should therefore provide tiered options according to differences in older adults’ physical functioning and cognitive abilities. By improving accessible facilities and safety measures and integrating active participation, passive viewing, and digital experiences, a multilevel and highly adaptable therapeutic landscape intervention system can be developed.

#### 4.3.3 Sociocultural experiential landscapes

Sociocultural experiential landscapes primarily facilitate interaction among older adults, nature, society, and other people through activities such as choral singing, landscape painting, workshops, and group cultural activities in parks. Compared with nature-based landscapes and structured intervention landscapes, their core emphasis lies in activity participation, cultural expression, and social interaction. Interest-based attraction, contextual immersion, and behavioral engagement generated through these activities can help alleviate loneliness and social isolation among older adults and strengthen their sense of group belonging. Rose and Lonsdale (2016) found that landscape-themed painting activities enhanced older adults’ sense of self-worth, social connectedness, and subjective well-being through visual expression, memory recall, and self-narration. Cai et al. (2023) indicated that collective singing in public landscape spaces helped promote psychological relaxation, positive emotions, social belonging, and group identity. Milligan et al. (2015) suggested that handicraft production, skill sharing, collaborative work, and everyday social interaction helped older adults maintain their previous work-related experiences, rebuild their sense of personal value, and strengthen social connectedness, belonging, and meaning in life.

Overall, sociocultural experiential landscapes represent a process in which spatial environments, social activities, cultural expression, and psychological restoration interact. Their advantage lies in their ability to become integrated into older adults’ daily lives in a non-medicalized manner and to promote emotional relaxation, social connectedness, and a sense of belonging through shared interests and group identity (Das et al., 2025a). However, existing studies have difficulty distinguishing the respective effects of the spatial environment, activity content, and group interaction. Moreover, participants are often volunteers or long-term participants, which may introduce selection bias (Skingley et al., 2016). In addition, because of the lack of control groups, standardized scales, and long-term follow-up, the actual effects, duration of benefits, and causal relationships remain difficult to determine (Milligan et al., 2016; Foettinger et al., 2022).

Therefore, in design practice, flexible spaces should be provided for activities such as painting, choral singing, and handicraft production, while seating, shade, and accessible facilities should be incorporated to support the sustained participation of older adults with different levels of ability. Future research should expand the sample size and geographical scope, pay greater attention to non-participants and those who withdraw during the intervention, and compare the effects of different cultural activities, as well as their frequency, duration, organizational support, and spatial accessibility. The applicability of imagined landscapes and digital landscapes for older adults with limited mobility should also be further explored.

### 4.4 Limitations

This study has several limitations. First, the search was restricted to English-language, peer-reviewed studies published between January 1, 2015, and October 19, 2025. Non-English-language studies, grey literature, and unpublished studies were not systematically included. Consequently, some relevant evidence published before the specified period or not yet publicly available may have been missed, and the review may be subject to language bias and publication bias. Because studies reporting positive or statistically significant findings are generally more likely to be published, retrieved, and included, the existing evidence may, to some extent, overestimate the positive effects of therapeutic landscapes on older adults’ mental health. Second, although this study expanded the scope of the database search, database coverage remained limited. Databases in the fields of nursing, biomedicine, and clinical research, such as CINAHL and Embase, were not searched; therefore, some relevant studies may still have been omitted. Third, substantial heterogeneity existed across the included studies in terms of research design, participant characteristics, therapeutic landscape types, intervention approaches, and the measurement of mental health outcomes. In addition, some studies did not report comparable effect sizes or complete statistical data. Therefore, a meta-analysis was not conducted, and the pooled effect size of therapeutic landscapes on older adults’ mental health could not be estimated. Finally, this study primarily focused on mental health outcomes such as depression, anxiety, stress, loneliness, and psychological well-being. Cognitive-related indicators, including cognitive functioning and cognitive impairment, were not included. Therefore, the conclusions of this study should not be directly generalized to the effects of therapeutic landscapes on the cognitive health of older adults.

## 5 Conclusion

This study followed the PRISMA 2020 guidelines and systematically searched for and included 21 relevant studies to review the evidence on the effects of therapeutic landscapes on older adults’ mental health. Regarding the general characteristics of the included studies, the existing research was geographically concentrated mainly in China, the United Kingdom, and the United States. Sample sizes varied considerably across studies, and sociodemographic information was insufficiently reported. In terms of measurement instruments and therapeutic landscape intervention approaches, the existing studies primarily used instruments such as depression scales and Smiley Face Scales, together with qualitative methods such as interviews and group discussions. The therapeutic landscape interventions examined in the included studies could be classified into three types: nature-based landscapes, structured intervention landscapes, and sociocultural experiential landscapes. The findings indicated that therapeutic landscapes had positive effects on older adults’ mental health, primarily by alleviating depression, anxiety, stress, and loneliness and enhancing psychological well-being. However, some studies also indicated that not all therapeutic landscapes consistently promoted mental health. Their actual effects were moderated by factors such as spatial accessibility, facility suitability, activity organization, seasonal and climatic conditions, and individual abilities. Further analysis showed that different types of therapeutic landscapes had differential effects on older adults’ mental health. Nature-based landscapes focused on landscape exposure and restorative experiences, structured intervention landscapes emphasized active participation and experiences arising from the intervention process, whereas sociocultural experiential landscapes highlighted social interaction, cultural expression, and emotional support. Overall, this field has established a certain international research foundation. Nevertheless, several limitations remain, including a limited volume of publications, uneven geographical distribution, substantial heterogeneity in research designs, and insufficient attention to gender and age differences. Future research should strengthen the accumulation of empirical evidence across diverse regions and sample types, promote the interdisciplinary integration of theories from multiple fields, and pay greater attention to the differing needs of older adults according to gender, age stage, health status, and sociocultural background. Further studies should identify heterogeneous effects across different therapeutic landscape types and groups of older adults and clarify the distinct pathways through which landscape exposure, landscape use, and landscape experience contribute to mental health outcomes.

## Data Availability

The original contributions presented in the study are included in the article/Supplementary material, further inquiries can be directed to the corresponding author.
