# Supplementary material for: The impact of therapeutic landscapes on the mental health of older adults: a systematic review
Source: Front Psychol. 2026 Aug 20;17:1898757. doi: 10.3389/fpsyg.2026.1898757 (PMC13538852; doi:10.3389/fpsyg.2026.1898757)
Supplement: Supplementary file 1 [file Supplementary_file_1.docx]

Supplementary Material

**Supplementary File 1.** Detailed search strategy

**Table S1.** Detailed search strategy

| **Database** | **Strategy** | **Results** |
| --- | --- | --- |
| Web of Science | ALL=((therapeutic landscapes OR healing garden)AND(mental health OR psychological health OR psychological well-being OR anxiety OR depression OR stress OR loneliness )AND (older adult” OR “older adults OR older person OR older persons OR older people OR elderly)) | 430 |
| SCOPUS | ("therapeutic landscapes" OR "healing garden")AND("mental health"OR "psychological health" OR "psychological well-being" OR "anxiety" OR "depression" OR "stress" OR "loneliness") AND ("older adult" OR "older adults" OR "older person" OR "older persons" OR "older people" OR "elderly") | 148 |
| JSTOR | ("therapeutic landscapes"OR"healing garden") AND ("mental health"OR "psychological health" OR "psychological well-being" OR "anxiety" OR "depression" OR "stress" OR "loneliness" ) AND ("older adult" OR "older person" OR "older persons" OR "older people" OR "elderly") | 167 |
| ScienceDirect | (“therapeutic landscapes” OR “healing garden”)AND("mental health"OR "psychological health" OR "psychological well-being" OR "anxiety" OR "depression" OR "stress" OR "loneliness")AND ("older adult" OR "older adults" OR "older person" OR "older persons" OR "older people" OR "elderly") | 662 |
| PubMed | (therapeutic landscapes) OR (healing garden) AND (mental health) OR (psychological health) OR (psychological well-being) OR (anxiety) OR (depression) AND (older adults) OR (older person) OR (older people) OR (elderly) | 139 |
| Apa PsyInfo | (Therapeutic Landscapes OR healing garden) AND (mental health) OR (psychological health) OR (psychological well-being) OR (anxiety) OR (depression) AND (older adults) OR (older person) OR (older people) OR (elderly) | 211 |

**Supplementary File S2**. JBI Risk-of-Bias Assessment Results

**Table S2.1.** Risk-of-Bias Assessment for Analytical Cross-Sectional Studies

| **N** | **Study** | **Q1** | **Q2** | **Q3** | **Q4** | **Q5** | **Q6** | **Q7** | **Q8** | **Score** | **percent(%)** | **Risk** |
| --- | --- | --- | --- | --- | --- | --- | --- | --- | --- | --- | --- | --- |
| 1 | (Dempsey et al., 2018) | 1 | 1 | 1 | 1 | 1 | 0 | 1 | 1 | 7/8 | 87.5 | Low risk |
| 2 | (Samadi-Todar & Hami.,2025) | 1 | 1 | 1 | 0 | 0 | 0 | 1 | 1 | 5/8 | 62.5 | Moderate risk |
| 3 | (Lin et al. ,2025) | 1 | 1 | 1 | 1 | N/A | N/A | 0 | 1 | 5/6 | 83.3 | Low risk |
| 4 | (Lu et al., 2021) | 1 | 1 | 1 | 1 | N/A | N/A | 1 | 1 | 6/6 | 100 | Low risk |
| 5 | (Zhang et al., 2021) | 1 | 1 | 1 | 1 | 1 | 0 | 1 | 1 | 7/8 | 87.5 | Low risk |
| 6 | (Hami et al., 2025) | 1 | 1 | 1 | 1 | 0 | 0 | 1 | 1 | 6/8 | 75 | Low risk |
| 7 | (Browning et al., 2019) | 1 | 1 | 1 | 1 | 1 | 1 | 1 | 1 | 8/8 | 100 | Low risk |
| 8 | (Guo et al., 2024) | 1 | 0 | 1 | 1 | 1 | 1 | 1 | 1 | 7/8 | 87.5 | Low risk |
| 9 | (Corley et al., 2021) | 1 | N/A | 1 | 0 | 1 | 1 | 1 | 1 | 6/7 | 85.7 | Low risk |

**Table S2.2.** Risk-of-Bias Assessment for Randomized Controlled Trials

| **N** | **Study** | **Q1** | **Q2** | **Q3** | **Q4** | **Q5** | **Q6** | **Q7** | **Q8** | **Q9** | **Q**  **10** | **Q**  **11** | **Q**  **12** | **Q**  **13** | **Score** | **percent（%）** | **Risk** |
| --- | --- | --- | --- | --- | --- | --- | --- | --- | --- | --- | --- | --- | --- | --- | --- | --- | --- |
| 1 | (Sia et al. ,2018) | 0 | 0 | 1 | 0 | 0 | 0 | 1 | 1 | 1 | 1 | 1 | 1 | 1 | 8/13 | 61.5 | Moderate risk |
| 2 | (Hao et al., 2024) | 0 | 0 | 1 | 0 | 0 | 0 | 1 | 1 | 1 | 1 | 1 | 1 | 1 | 8/13 | 61.5 | Moderate risk |
| 3 | (van Houwelingen-Snippe et al., 2023), Study 2 | 0 | 0 | 0 | 0 | 0 | 0 | 1 | 1 | 1 | 1 | 1 | 1 | 1 | 7/13 | 53.8 | Moderate risk |

**Table S2.3**. Risk-of-Bias Assessment for Qualitative Research

| **N** | **Study** | **Q1** | **Q2** | **Q3** | **Q4** | **Q5** | **Q6** | **Q7** | **Q8** | **Q9** | **Q10** | **Score** | **percent（%）** | **Risk** |
| --- | --- | --- | --- | --- | --- | --- | --- | --- | --- | --- | --- | --- | --- | --- |
| 1 | (Zumelzu et al., 2025) | 1 | 1 | 1 | 1 | 1 | 0 | 0 | 1 | 1 | 1 | 8/10 | 80 | Low risk |
| 2 | (Rose & Lonsdale.,2016) | 1 | 1 | 1 | 1 | 1 | 0 | 0 | 1 | 0 | 1 | 7/10 | 70 | Low risk |
| 3 | (Milligan et al., 2015) | 1 | 1 | 1 | 1 | 1 | 0 | 0 | 1 | 1 | 1 | 8/10 | 80 | Low risk |
| 4 | (Zatovicová & Kubánová, 2024) | 1 | 1 | 1 | 1 | 1 | 0 | 0 | 1 | 1 | 1 | 8/10 | 80 | Low risk |
| 5 | (Cai et al., 2023) | 1 | 1 | 1 | 1 | 1 | 0 | 0 | 1 | 1 | 1 | 8/10 | 80 | Low risk |
| 6 | (Finlay et al., 2015) | 1 | 1 | 1 | 1 | 1 | 0 | 0 | 1 | 1 | 1 | 8/10 | 80 | Low risk |
| 7 | (Finlay., 2018) | 1 | 1 | 1 | 1 | 1 | 0 | 0 | 1 | 1 | 1 | 8/10 | 80 | Low risk |
| 8 | (Milligan et al., 2021) | 1 | 1 | 1 | 1 | 1 | 1 | 0 | 1 | 1 | 1 | 9/10 | 90 | Low risk |
| 9 | (van Houwelingen-Snippe et al., 2023), Study 1 | 0 | 1 | 1 | 1 | 1 | 0 | 0 | 1 | 1 | 1 | 7/10 | 70.0 | Low risk |

Table S2.4. Risk-of-Bias Assessment for Quasi-Experimental Studies

| **N** | **Study** | **Q1** | **Q2** | **Q3** | **Q4** | **Q5** | **Q6** | **Q7** | **Q8** | **Q9** | **Score** | **percent（%）** | **Risk** |
| --- | --- | --- | --- | --- | --- | --- | --- | --- | --- | --- | --- | --- | --- |
| 1 | (Akindejoye et al., 2025) | 1 | 0 | N/A | N/A | 0 | 1 | 1 | 0 | 1 | 4/7 | 57.1 | Moderate risk |
